# Intended, Unintended, Unanticipated? Consequences of Social Distancing Measures for Nursing Home Residents During the Covid-19 Pandemic

**DOI:** 10.1177/23333936231176204

**Published:** 2023-05-28

**Authors:** Laila Tingvold, Jill-Marit Moholt, Oddvar Førland, Frode Fadnes Jacobsen, Oscar Tranevåg

**Affiliations:** 1 Norwegian University of Science and Technology (NTNU), Gjøvik, Norway; 2 UiT The Arctic University of Norway, Tromsø, Norway; 3 Western Norway University of Applied Sciences, Bergen, Norway

**Keywords:** COVID-19, pandemic, nursing home, social distancing measures, residents, Norway

## Abstract

During the outbreak of the COVID-19 pandemic, Norwegian health authorities introduced social distancing measures in nursing homes. The aim was to protect vulnerable residents from contracting the potentially deadly infection. Drawing on individual interviews with nursing home managers and physicians, and focus groups with nursing staff, we explore and describe consequences the social distancing measures had on nursing home residents’ health and wellbeing. The analysis indicates that most residents became socially deprived, while some became calmer during the nursing home lockdown. Nursing home staff, physicians and managers witnessed that residents’ health and functional capacity declined when services to maintain health, such as physiotherapy, were put on hold. In conclusion, we argue that although Norwegian health authorities managed to keep the infection rates low in nursing homes, this came at a high price for the residents however, as the social distancing measures also negatively impacted their health and wellbeing.

## Introduction

When the World Health Organization (WHO), on March 11th, 2020, redefined the outbreak of COVID-19 from an epidemic to an out-of-control pandemic, health authorities in many countries developed and implemented measures specifically to prevent the infection from reaching nursing home (NH) residents. NHs typically provide care for one of the most vulnerable populations in society, including older people with physical and cognitive impairments and residents with chronic medical conditions. In Norway, recommendations and procedures to prevent the spread of the infection were issued by the Norwegian Directorate of Health and the Norwegian Institute of Public Health. In NHs, measures were directed toward staff regarding the use of equipment, personal protection gear and work procedures, as well as instructions regarding which test criteria and symptoms should invoke testing among staff and residents. Other measures were addressed more directly at protecting and shielding the residents, to attempt to stay ahead of the infection and hence reduce the likelihood of residents contracting COVID-19 (Norwegian Directorate of Health, 2022; Norwegian Institute of Public Health, 2022). Overall, in this latter category with a focus on social distancing, four main measures were implemented: (a) isolation of the residents in their individual rooms or wards to prevent close contact with others and reduce the likelihood of contracting and spreading the virus within the NH, (b) shutting down cultural and social activities run by NH staff themselves or by outside volunteers, (c) putting physiotherapy services and personal care services (such as hairdressing, podiatrists) on hold, and (d) implementing a visiting ban, restricting access for relatives to visit their family members in NHs (Jacobsen et al., 2021).

A substantial body of research exists on how COVID-19 affected residents and staff in NHs. Several quantitative studies have concluded that the implications of the pandemic for NH residents were severe in terms of illness and death (Thompson et al., 2020), loneliness and reduced wellbeing (Levere et al., 2021; Van der Roest et al., 2020), worsening of physical health (Levere et al., 2021) and increased use of medication, for example, psychotropics and antidepressants (Campitelli et al., 2021; Stall et al., 2021). Previous qualitative studies including residents and/or their family members support several of these findings and show that the residents suffered from loneliness and unmet social needs (Lood et al., 2021; Noten et al., 2022), as well as grief, stress and deterioration in physical abilities and cognitive functioning (Paananen et al., 2021). Other qualitative studies have examined the impact of the pandemic on health and wellbeing among NH staff, as well as on working practices in NH (Hanna et al., 2022; Hoedl et al., 2022; Sarabia-Cobo et al., 2021; Yau et al., 2021). To our knowledge, there is, however, a lack of qualitative studies of the perspectives of NH staff, physicians and managers (from now on referred to as healthcare personnel (HCP) on how residents were impacted by the social distancing measures introduced. These perspectives are important because HCP in NHs are a primary source of information, as they were close to the residents and have important observations and perspectives to share. An important aspect to address concerning this knowledge gap is related to the perspectives of HCP on the consequences of the social distancing measures for Norwegian NHs residents’ health and wellbeing—when these restrictions were used as purposive and formally organized actions to prevent spreading the COVID-19 virus in Norwegian NHs.

### Norwegian Nursing Homes

Norway has a universalistic, nationalized health care system financed through taxes which includes NHs at the local municipal level. NHs are primarily meant for frail older adults with advanced dementia or extensive functional decline. Access is in principle equal, regardless of income, social status and family situation. In Norway, NHs are the final home for many of the oldest citizens with advanced chronic illnesses and multiple diagnoses (Næss et al., 2013). According to Statistics Norway, there were approximately 40,000 beds in Norwegian NHs in 2021 (Statistics Norway, 2022), most on long-term stays and one-third on short-term stays, for example, patients admitted to a municipal acute care unit or discharged from hospitals, patients on rehabilitation therapy, and those waiting for a permanent place in an NH (Melby et al., 2019). The average length of long-term stay after NH admission is around 2 years. About 50% of NH residents die within a year (Kjelvik & Jønsberg, 2017). Over the last decade, NH residents’ healthcare needs have increased, and they often have multiple diagnoses requiring treatment (Melby et al., 2019). These residents on average take 8 medicines, and approximately 8 out of 10 residents have a dementia diagnosis (Gulla, 2018). Their overall high burden of disease makes them more vulnerable and susceptible to infectious diseases such as COVID-19 (Jacobsen et al., 2021). All NHs have at least one physician who systematically follows up on medical treatment and performs medical examination of the residents. The physician also assesses residents’ ability to consent, capacity to handle their own funds and their potential need for a legal guardian. Moreover, the physician has a supervisory role as to instructing the general staff on medical matters.

Due to the declining level of functioning among residents, increased requirements for documentation, and the transfer of responsibilities from specialist care services to the municipal level of healthcare, the overall pressure on NH staff is growing (Gautun & Syse, 2017; Jacobsen et al., 2021; Romøren, 2018). It is reasonable to assume that the increasing number of short-term stays in NHs, and the increased disease burden and the number of deaths, have generated a need for more medically directed work in NHs during the last decade (Melby et al., 2019). In this context, formal skills are increasingly needed, but recruiting qualified staff to work in NHs often fails. There is a widespread use of part-time temporary and zero-hours contract positions. The sick-leave percentage is high, and many NHs rely on substitute workers or unqualified assistants to cover shifts 24/7 (Orupabo & Nadim, 2020; Tingvold & Fagertun, 2020). In the Norwegian context, the sector is also characterized by low wages, heavy workloads, and demanding shift work. About 25% of the workforce are low skilled, defined as workers without formal education (Tjerbo et al., 2012). From an international comparative perspective, the staffing levels and formal staff competence in Norwegian NHs are, however, relatively high (Harrington & Jacobsen, 2020; Harrington et al., 2012).

Another concern has been the insufficient psychosocial care of NH residents (Bergland & Kirkevold, 2006; Kjøs & Havig, 2016). Studies show that basic care, such as morning care, is of a relatively high quality, while the quality and volume of physical and social activities in these facilities are lower and in need of improvement (Kirkevold & Engedal, 2006; Kjøs & Havig, 2016). However, the quality of care varies, and the Norwegian government launched a quality reform (Norwegian Ministry of Health and Care Services, 2018) and a safety standard for NHs (Norwegian Ministry of Health and Care Services, 2021). The latter is a work tool developed for NH managers, which aims to ensure dignity and safety among the residents and to reduce variations in the quality of care.

### Objective

This paper is based on data from the project *Experiences with COVID-19 in Norwegian nursing homes*, commissioned by the Norwegian Corona Commission in the autumn of 2020. The main objective of the project was to obtain overall knowledge about how HCP in NHs and residents’ family caregivers, experienced and handled the COVID-19 pandemic in Norwegian NHs during the first 9 months of the pandemic (Jacobsen et al., 2021).

Based on further in-depth analysis of the data of HCPs’ experiences, the objective of this paper is to critically explore and describe the perspectives of HCP’ in Norwegian NHs in relation to the consequences the social distancing measures had on NH residents’ health and wellbeing.

## Methods

### Research Design

A qualitative exploratory design was employed as this approach is advantageous when investigating underlying processes of a distinct phenomenon of which we have limited knowledge (Polit & Beck, 2010). A case study based on the epistemology of Gadamer’s philosophical hermeneutics (2004) was chosen. Case studies provide the opportunity for detailed investigations of a situation often based on several different types of data sources (Yin, 2013), whilst the epistemology of hermeneutics emphasizes how the interpretive movement of the hermeneutic circle is fundamental in developing new understanding of the essence of a specific phenomenon (Gadamer, 2004). Based on the hermeneutical foundation, the thematic analysis (TA) approach described by Braun and Clarke (2006, 2019, 2022) was used in the data analysis process. The TA approach offers qualitative researchers flexibility in terms of the philosophical assumptions underpinning the particular iteration of TA. Qualitative researchers from different epistemological positions searching for themes or patterns in their data can therefore use this approach, and we found it most helpful in establishing a structure for our hermeneutical data interpretation.

### Preunderstanding

To increase transparency (Hiles, 2008) and study trustworthiness (Guba et al., 1994; Lincoln & Guba, 1985; Polit & Beck, 2010), as well as to help the reader identify the interpretive context of the research, researchers’ preunderstanding of the study should be outlined (Gadamer, 2004). All authors have professional background in nursing or social sciences, and they hold extensive experience from research in nursing homes and long-term care. In this study, each researcher first endeavored to identify their own preconceptions through individual reflection. Thereafter, all authors had reflective dialogs about our preconceptions of what possible consequences the social distancing measures could have had for the NH residents. We expected to find that HCP who implemented these measures could describe observed side effects of the measures, that is, other and additional consequences than the prevention of spreading the COVID-19 virus in NHs. We assumed that the implemented social distancing measures also could have consequences for the residents’ health and wellbeing; for example if residents were isolated in their rooms—no longer allowed to maintain their social contact with others, and if the measures in some ways resulted in reduced quality of nursing care and medical care. We were also open for the possibility that HCP could describe positive consequences of the implemented social distancing measures. Along with this, the previous research findings presented in the introductory paragraph above, also formed part of our preunderstanding.

### Participants and Recruitment

The municipalities and the NHs were strategically selected to obtain variation in the cases regarding population size, infection pressure and incidence of COVID-19-related deaths. The five included NHs were located in different municipalities—one in each of the five former health regions: Northern, Central, Western, Southern, and Eastern Norway. The smallest municipality had a population of 3,000 inhabitants and the largest 780,000. More details of the included NHs are presented in Table 1.

**Table 1.** Details on the five Nursing Homes and Study Participants.

Nursing homes (NH)				Study participants		
	Location	No. of staff	No. of residents	No. of staff (focus groups)	No. of physicians (individual interviews)	No. of managers and ward managers (individual interviews)
NH 1	Urban	300	140	8	1	2
NH 2	Urban	170	90	4	2	2
NH 3	Rural	25	25	3	1	1
NH 4	Semi-urban	150	130	6	1	2
NH 5	Rural	20	21	5	0	1
Total		656	406	26	5	8

Initially, we contacted chief managers within the municipal healthcare administration to inform them about the project and to obtain permission to include the selected NHs in the study. The chief managers appointed NH managers as contact persons who further assisted the research team in the recruitment of participants and in the collection of vital documents. On behalf of the research team, the NH managers forwarded information letters to potential participants among NH ward managers, physicians and staff. The inclusion criterion was: Being an NH staff member, physician or manager who had part- or full-time employment in the NH during the outbreak of the COVID-19 pandemic. HCP who gave their informed consent to participate signed the consent form, and the research team then contacted them to schedule a time and place for data collection. The sample included 13 registered nurses, 10 auxiliary nurses, two nursing students and one nursing assistant. Most of them had considerable experience within the field of long-term care for older people—on average they had worked for 10 years in their respective NH. Further, the sample included five NH physicians, five NH managers, and three ward managers. The physicians had been in their current positions between 1 and 12 years. Of the eight NH managers and ward managers, seven were registered nurses and one was a social worker. On average they had been in their current positions for 7 years. They had between 16 and 40 years of work experience from long-term care for older people, and six of them had specialization within management, coaching, geriatric or psychiatric care.

### Data Collection

We conducted one focus group interview (Halkier, 2008) with the NH staff in each of the five NHs and semi-structured individual interviews (Brinkmann & Kvale, 2015) with NH managers. We further conducted semi-structured individual interviews with physicians in four of the five nursing homes. Due to large variations in the coverage of physicians in NHs (Melby et al., 2019) and the limited number of eligible NH managers, we considered individual interviews as most appropriate for these occupational groups. All five authors were responsible for conducting interviews in their respective regions. Individual interviews were carried out by one researcher, while the focus groups were performed by two researchers, a moderator and a co-moderator, in all NHs except one. Notes were taken both during the individual interviews and the focus groups. All researchers transcribed their own interviews.

All researchers used the same semi-structured interview guide that was developed based on the aims of the main project, previous knowledge about the outbreak of the pandemic in NHs, as well as thorough discussions between the members of the project group. In the interviews, the participants were initially encouraged to talk about topics related to the outbreak of the pandemic, for example, how they were prepared for a pandemic, and their knowledge about and use of infection control plans. Next, we asked the participants to reflect on how the pandemic was handled in the NHs. Relevant topics were implementation of infection control measures, the effectiveness of the measures, prioritizations of tasks, how the residents care needs were met, unmet care needs, collaboration and communication with the residents’ family members as well as consequences of the pandemic.

The interviews were carried out during the last week of November and the first weeks of December 2020, that is, about 9 months after the pandemic outbreak. The focus group interviews with the staff, consisting of 3 to 8 participants, were performed in all five NHs. Each interview lasted from 65 to 90 min. The individual semi-structured interviews with NH managers, ward managers and physicians lasted from 20 to 70 min. Due to the infection danger when the interviews took place, only one NH admitted the researchers physically to the NH to carry out the data collection there. In four NHs, interviews were conducted using the digital communication platform Teams or by telephone. The interviews were conducted in Norwegian. Quotes used in the article were translated by the first author and carefully checked by a professional bilingual proofreader. All interviews were audiotaped with the permission of the participants. More details about the data collection and participants are shown in Table 1.

### Data Analysis

As addressed by Braun and Clarke (2022, p. 12) research questions sometimes “evolve through the course of the research” as more detailed insight is produced. This was also the case for this study. In the beginning of the project, we sought to understand how HCP experienced and handled the situation during the first 9 months of the pandemic. During our further in-depth analysis, we became increasingly aware of how the HCP also addressed that the social distancing measures also had other consequences for the residents than the prevention of the spreading of COVID-19 virus in the NHs. Based on Gadamer’s hermeneutical philosophy (2004) and the thematic analysis approach of Braun and Clarke (2006, 2019, 2022) we therefore conducted further analysis of the collected data to gain new understanding also of this specific topic.

In line with first phase of the TA approach—*familiarizing yourself with the data* (Braun & Clarke, 2006), we as a research team began the interpretive hermeneutic process (Gadamer, 2004) with individual reading of the transcribed interview texts. By reading the texts several times each of us developed an overview of the data. Each new interview text made previously explored interview texts more understandable, while adding a new initial meaning of their own. Being aware of own preunderstanding and of how preunderstanding in hermeneutical interpretation can lay foundation for new and expanded understanding we emphasized being explorative and open, and not conclusive in our initial reading to familiarize with the data. In this process we arranged research team meetings, in which we shared and discussed our initial ideas and preliminary understanding of how the texts conveyed the HCP’ perspectives of possible consequences of the social distancing measures—on NH residents. Being familiarized with the data, we moved on to *generating initial codes* (Braun & Clarke, 2006, 2019), To identify a feature of the data and organize our data into meaningful groups, each researcher performed the initial coding of three to five interviews. All data across the interview texts that we perceived as possible elements giving meaning to the phenomenon—consequences of the social distancing measures of NH residents, were given an initial code. In this process we emphasized what Braun and Clarke (2019, p. 594) describe as “a continual bending back on oneself—questioning and querying the assumptions we are making in interpreting and coding the data.” In hermeneutic epistemology questioning and reassessing one’s own interpretation of the meaning of a text is a vital circular process of growing ideas termed the hermeneutic circle (Gadamer, 2004). During our interpretation process of generating initial codes, as well as in the later phases of generating themes, reviewing themes, and defining and naming themes, we emphasized the use of the hermeneutic circular movement. As researchers we went back and forth in each interview text to investigate the text parts, and the text as a whole—continuously questioning our preliminary understanding and seeking for other possible interpretations while aiming to identify the substance of the text and prevent our preunderstanding from concealing its essence.

Having identified our initial codes, we then searched for patterns in the codes that were related to our research aim. By doing so we initiated the process of *generating themes* (Braun & Clarke, 2006, 2019), in which we had focused attention on identifying interesting items in the data that we believed could form the basis of repeated patterns, that is, potential themes across the data set. Following the hermeneutic circle movement described above, we sorted the various codes into potential themes and collated all relevant coded data extracts within the different preliminary themes, using tables as tools for organizing the data analysis.

To develop a deeper understanding of the essence of the set of candidate themes, a further refinement is needed. In the process of *reviewing themes* (Braun & Clarke, 2006, 2019) we therefore continued to explore the coded extracts of each theme to evaluate whether they formed part of a coherent pattern. Coded extracts that we found coherent were retained within the theme, while those perceived as incoherent were either moved to other identified themes or to newly created themes, or they were discarded from the analysis. To consider the validity of the reviewed themes, we had focused attention on exploring how accurately they reflected the meaning in the data set as a whole. Again, using the hermeneutic circle movement was of great help for us—this time in our exploration and understanding of whether the refined themes clearly conveyed the meaning of the data set as a whole. In this process we also emphasized to further delimit the themes’ working titles, accordingly.

We then moved on to *defining and naming themes* (Braun & Clarke, 2006). In this process we maintained to refine the themes to be able capture the essence of each theme, aiming to describe our interpretations of the HCP’ perspectives as clearly as possible. On this basis it was possible for us to formulate the content of each theme in a in a few summary sentences. These concise formulations were then used to develop the theme titles—from working titles to final titles of the themes (Table 2).

**Table 2.** An Example From Phases in the Analysis Process.

**Familiarizing with the data—data extract** *“They [residents] were restless, this was unusual for them. Before. . . they could see when we [staff] were talking . . . but when we* *started wearing personal protective equipment . . .they did not understand what we were saying”* (Nurse, NH1)		
**Generating initial codes** a. Stressful changes for residents b. Miscommunication and lack of connection		
**Generating themes** - New routines were implemented overnight - Protective gear worn by staff hampered talking and connecting with residents - Residents were restless and strove to understand the changes - Difficult, and especially hard for residents with cognitive impairment and wandering behavior to comply to the new routines regulating contact and movement - Use of sedatives to curb challenging behavior in stressed and anxious residents	**Reviewing themes** Sudden and intrusive changes regulating physical movement and socialization in the nursing homes caused stress and confusion in many residents. Many residents strove to make sense of the new situation, especially residents with cognitive impairments were upset, and could not comply to new restrictions.	**Defining and naming themes** Confusion and communication challenges

## Theoretical Framework

According to Gadamer (2004) the hermeneutic approach also involves developing a theoretical understanding of the phenomenon under investigation. In our data analysis process, the theoretical perspectives of Merton (1936) on *consequences of purposive actions* were found to be a fruitful theoretical basis for the empirical-theoretical discussion. In his seminal work—*The unanticipated consequences of purposive action*, Merton (1936, p. 895) defined consequences as “those elements in the resulting situation, which are exclusively the outcome of the action, that is, those elements which would not have occurred had the action not taken place.” It is beneficial to distinguish between intended (planned) and unintended (unplanned, nevertheless sometimes anticipated) consequences of purposive social actions. Intended consequences are desirable to the actor but may in some cases be regarded as undesirable, adverse and negative to an outside observer. While the actor is aware of the intended consequences because they are planned, the unintended consequences are often surprising and more difficult to identify and recognize. Merton (1936) primarily uses the term “unanticipated consequences” (unplanned, unforeseen, and surprising). Importantly, an action may have intended, unintended and unanticipated consequences. In a later work, Merton (2016) uses and adapts the expression “latent functions” related to actions and activities that are neither recognized nor intended, in opposition to “manifest functions,” which refer to directly observable, expected and intended actions. All actions create both manifest (immediately visible) consequences and latent (hidden or unknown) consequences, and it is essential to study both.

When it comes to unintended consequences, de Zwart (2015) distinguishes this concept from unanticipated consequences, with reference to Merton’s (1936) paper. De Zwart is critical of the widespread conflation of these two concepts, a conflation Merton himself contributed to later in his principal work, *Social Theory and Social Structure* (Merton, 1968), a landmark publication in sociology. De Zwart’s point is that unintended consequences are not necessarily unexpected and surprising and therefore must be distinguished from unanticipated and unforeseen consequences. This is an important nuance and distinction and in accordance with Merton’s paper published in 1936.

Further, the concept of unanticipated consequences is normatively neither positive nor negative, even though it is often associated with undesirable and adverse outcomes. It may relate to an unexpected drawback of an intended and desired (policy) action but sometimes also to an unexpected benefit. In some cases, the outcome may be described in the first place as a positive outcome, while it later turns out to have unanticipated negative consequences. Thus, these concepts are multifaceted, with diverse meanings.

In short, purposive actions comprises three distinct different categories of consequences:

- Intended (planned) consequences
- Unintended (unplanned) but not unanticipated/surprising consequences (calculated risk)
- Unintended (unplanned) and unanticipated/unforeseen/surprising consequences

## Ethical Considerations

The project was approved by the Norwegian Centre for Research Data (323341) and was carried out in accordance with research ethics guidelines described in the Declaration of Helsinki (World Medical Association, 2013). The managers of the five NHs gave their consent for NH to participate in the study before recruiting the study participants. All participants gave written informed consent to participate in the study. The participants were informed of their right to withdraw from the study without stating a reason, and data was managed with respect to the anonymity and confidentiality of all participants. All interviews were anonymized during transcription and securely stored and processed through a data platform with multi-factor authentication.

Based on analysis of the empirical data, we identified three main themes related to consequences of the social distancing measures—for the residents: Confusion and communication challenges; Social deprivation and loneliness, and; Reduced healthcare services leading to deterioration of health condition.

## Confusion and Communication Challenges

At the outbreak of the pandemic, HCPs observed that residents generally struggled to comprehend what was happening. Many residents could neither understand the meaning of the word “pandemic” nor the reasons for the social distancing measures. Some residents could follow national television news or communicated by telephone with family members who helped to make sense of the measures—explaining the changes it caused in daily life in the NH. Staff described how some of the residents adapted quite well to the situation, while many, especially residents with cognitive impairment, were confused and stressed. They were often in need of repeated explanations and sensemaking of the altered situation. A ward manager stated that residents were particularly confused about why their family members had stopped visiting them:

*. . .every day we reminded them* [the residents] *that now there was a pandemic that prevented relatives from coming to visit, and we had to repeat that information. So, we could reassure them that it was not because . . . their family did not want. . .to visit them*. (Ward manager, NH 1)

Several residents were confused and frustrated about being isolated in their ward and in their own rooms. Isolation in the resident’s room took place if staff observed symptoms coherent with COVID-19. In a NH with an outbreak of the infection in one of the wards, the staff tried to isolate the eight residents in their own individual rooms. However, this turned out to be difficult because two of the residents with dementia were so-called “wanderers”, having no understanding of the new rules and arrangements.

In this setting, residents with wandering behavior were a big challenge for the staff, as they were considered more likely to spread the infection. The manager in one NH explained she had to isolate the other residents from the wanderers because they could more easily accept isolation and stay inside their own rooms. A NH physician reflected on how harmful it was for residents with wandering behavior to be isolated:

*You cannot constantly hold still a person with dementia who is walking. . . and who does not know when he or she is inside his or her own room and when he or she is in someone else’s room! It is very harmful, I think, to do so* [isolate], *so it is a totally different situation* [than isolating residents who are cognitively lucid]. (Physician, NH 2)

HCP stated that some of the residents, who were very confused and anxious, were given larger doses of sedative medication than usual, to reduce stress and to curb episodes of challenging behavior. This made it easier to isolate residents, but did also rise some serious dilemmas as highlighted by this physician:

*There are many dilemmas, there are many things to discuss. It is also about this: Can you isolate old people who do not understand? What are the ethical aspects? And. . . can you test them. . . what about those who do not give consent? There has also been a lot of discussion of both testing and isolation in relation to consent, coercion – how to handle these things in practical terms.* (Physician, NH 2)

At the outbreak of the pandemic, staff were obliged to start wearing personal protective gear. Some members of staff explained that residents were uneasy and anxious when staff entered their rooms in personal protective gear, as one of them put it; “*looking like aliens in moon suits.”* The protective gear, like visors and face masks, also significantly hampered direct verbal communication with the residents, and misunderstandings could easily occur. A nurse said:

*They* [residents] *were restless, this was unusual for them. Before. . . they could see when we* [staff] *were talking. . . but when we started wearing personal protective equipment, such as face masks, visors, gloves, . . . and we always wore this protective gear . . . they [residents] did not understand what we were saying! There are many residents who have hearing loss and, in that situation* [of difficult communication], *we could not just take off the personal protective equipment and satisfy their need for contact*. (Nurse, NH 1)

The staff tried to facilitate contact and communication between the residents and their families, using digital devices such as tablet computers. While some residents benefited from this communication to some extent, it caused confusion and distress among several of the residents with cognitive impairment and, in many cases, proved useless. Residents had thrown away the tablets and were unable to connect. Staff explained that some residents had problems recognizing their family members when they visited physically and claimed that it didn’t get any easier when they were on a tablet. This made staff having to be present and help when tablets were in use:

*Some people* [residents] *think the tablet computers are a bit strange. . . we have had both laughter and crying. . . and some residents are trying to give hugs to people on the screen. . . they don’t understand the technology, so they need some help and that we are there assisting*. (Manager NH 3)

In summing up this section, the HCP witnessed that several of the residents experienced confusion and communication problems because of the social distancing measures. They observed that residents with dementia or cognitive challenges particularly struggled to understand the new situation and to comply with what, for them, were new incomprehensible rules and regulations.

## Social Deprivation and Loneliness

At the outbreak of the pandemic, all physical, social and cultural activities organized by the NH staff or volunteers from external organizations were stopped. In addition, a ban on visiting was introduced, and family members were no longer allowed to be physically present in the NH facilities. These restrictions prevailed for several months. Each of the included NHs in this study adhered to the national guidelines and isolated residents with the intention of shielding them from infection and minimizing their likelihood of contracting the infection. Our data from interviews with HCP, including NH managers, indicate that NH 1 and NH 2 were more severely affected by COVID-19 infection. At the time when this study took place, both NHs experienced that some residents died as a result of the infection, and that several other residents, and staff, also experienced being infected. The three other NHs had few or no cases at all. Two NHs (NH 4 and NH 5) were located in municipalities with very low infection rates. A manager in one of these municipalities without infection stated:

*. . . everything was closed and there was a visiting ban, relatives were not allowed to come. We stopped all physiotherapy programs in the wards, all the activities. . . religious services, visits by volunteers. . . yes everything was stopped. It was, of course, reopened when national recommendations allowed it. We don’t have any local policies that go beyond the national ones. We follow the national guidelines.* (Manager, NH 5)

HCP in NHs implemented the strict regulations sometimes hesitantly. A physician said:

*I would say that the most important thing that can prevent an outbreak of the virus in a nursing home is absolutely terrible. . . but it is that isolation.* (Physician NH 2). However, staff were afraid the virus would spread and harm old frail patients: *We have people older than hundred years here. And. . . if people* [were to start] *dying from the virus* [COVID-19], *that is absolutely terrible. So we have been working to prevent that*. (Nurse, NH 4)

Staff and managers responsible for residents’ daily care claimed however that some residents adapted surprisingly well to the new restrictions. Some appeared more relaxed or calm, and it seemed like some residents valued fewer activities and people present on the ward. One nurse said for example: *For an infected resident who has . . . a mental disorder, he actually felt better from . . . being sheltered in the room and being more to himself. . . .and having more peace around him.* (Nurse, NH 1)

However, staff in all five NHs observed that the majority of the residents became very lonely and suffered. A nurse stated:

*The residents have been by themselves a lot. When the pandemic hit, all residents had to stay in their rooms. 24/7! Including those who were not infected. . . and there was a total ban on visiting. The residents were alone, and it had consequences for their psychosocial health.* (Nurse, NH 1)

Staff described the problem of public health authorities becoming too concerned about protecting the residents’ health and wellbeing; if taken too far, they stated, it would hamper the residents’ quality of life very negatively and give them a less meaningful life. However, staff claimed that the isolation hit the residents unequally; those who were used to having an active social life with daily visitors prior to the pandemic were more harmed by the isolation.

In another NH, a nurse also differentiated among groups of residents in how they were affected by the isolation. On her ward, they experienced three rounds of isolation during the first 9 months of the pandemic. She stated:

*I think that some of the residents became depressed. They were resigned! Some handled it okay. It might be easier for those with cognitive impairment compared to those who are cognitively lucid and can count the days. Because for them. . . the longing and missing of family is substantial.* (Nurse, NH 1)

Managers in some of the NHs tried to replace the loss of activities by organizing small concerts, reading aloud or arranging exercises by ward. Some received funds from the regional health authorities to support such activities. Staff working on wards with infection did not work elsewhere in the NH but stayed in the ward throughout their shifts. They claimed that this change helped facilitate a better collegial milieu on the wards and closer contact with the residents:

*Now we come to work and are with the residents. . . on the ward until we go back home. . . . it is positive for the residents that we are much closer to them. We used to go out of the ward to have lunch* [prior to the pandemic], *but now we are on the ward* [with the residents] *and we have continued to do that.* (Auxiliary nurse, NH 2)

Staff also expressed special concern for terminally ill residents who, due to the visiting ban, could not have their families present as much as they wished for in the last days of their lives. Only residents in the terminal phase were allowed to have visitors, however it could be hard to predict when a resident was dying and how to facilitate visiting in time. A NH manager said:

*It has been and still is difficult, I think, because we know that these patients are in . . . the final phase of life . . . and then . . . witnessing that, what would help them most, and that would be right, even if they are depressed and passive, is being with their loved ones. Not being able to offer them that, that’s a big problem, right?* (Manager, NH 1)

About 2 to 3 months after the outbreak of the pandemic, residents were allowed visitors, providing that they were healthy and followed the prevailing infection control measures, like use of masks, respecting rules concerning the restricted number of visitors, carrying out visits in the residents’ own rooms, and avoiding all physical resident-visitor contact. In some of the NHs, staff noticed that family members did not resume the same frequent visiting pattern as prior to the pandemic. Generally, staff noted fewer and shorter visits. A nurse stated:

*They* [the family members] *come now and then . . . There are somewhat less visits, they did not come as frequently as before. Some, who previously visited the residents daily . . .they now come every other day or once a week. The residents think this is sad because they were used to the daily visits. It seems like the residents who understand the situation have adapted to it (. . .).* (Nurse, NH 5)

In summing up, HCP experienced that most of the residents became lonely and depressed when social distancing measures were put in place. Residents used to be socially active, and those who “*could count the days”* seemed to be hit hard by the restrictions. Some residents appeared to be calmer by having less activities around them. In some NHs staff did organize ward-based social activities to connect colleagues and residents, and to reduce some of the negative consequences of the social distancing measures introduced during the pandemic. HCP were particularly concerned about the consequences for residents living their final days and for those that were dying alone.

## Reduced Healthcare Services, Leading to Deterioration of Health Condition

According to some nurses, residents received less frequent medical attention from NH physicians during the pandemic. Nurses often replaced the physician in the medical visits and discussed observations and treatment interventions with the physicians by telephone. A nurse explained the altered medical follow-up within the NH, like this:

*. . .there was a shift in roles – nurses were given new and extended responsibilities and performed more medical tasks, while the auxiliary nurses carried out some of the nurses’ tasks.* (Nurse, NH 5)

In a NH with COVID-19 infection, staff were permitted to stay in residents’ rooms for a maximum of 15 min at a time. This caused difficulties in carrying out certain care tasks. Helping residents to shower was one such task reported to be frequently omitted, due to the time limitation:

*We* [staff] *had 15 minutes we could stay inside the room at a time, then we had to go out, and we were very strict about that. . . 15 minutes and no more. . . so, no one got a shower for four weeks. . . They got a full body wash when we were two staff members in the room, but we could not shower them. . . . If you are going to maintain it* [giving a shower] *100%, then it’s not possible to have only 15 minutes.* (Auxiliary nurse, NH 2)

Physiotherapy services were put on hold, and residents with diagnoses that required regular physical exercises and activation were therefore negatively affected by the cancelation of therapy. A NH physician claimed that many residents suffered from the social distancing measures not only because there had been fewer visits, contact and stimuli, but also because the cessation of physiotherapy services had led to deterioration in the residents’ physical health:

*They* [residents] *have . . .been depressed and have deteriorated physically because they have not received physiotherapy. And that is a big concern, and it still is. In other words, in geriatrics, we are very concerned with movement and physiotherapy and exercising to maintain functions, and also to avoid things like oedema. . .avoid . . . provide for mucus mobilization that may otherwise increase the risk of pneumonia, etc. So, I have been . . . more concerned about the passivity.* (Physician, NH 1)

In summing up, due to the social distancing measures, fewer health- and care services were offered during this first part of the pandemic. In some cases, nurses conducted medical visits that the physician normally would have performed, and auxiliary nurses took over tasks previously mainly performed by nurses. Physiotherapy services were put on hold, and residents’ physical health was negatively affected when no longer receiving exercises to maintain mobility and physical functions.

## Discussion

Our findings of consequences of the social distancing measures for Norwegian NHs residents’ health and wellbeing are based on experiences and perspectives of HCPs who were close to the NH residents. We found that they had important observations and perspectives to share, however, no sex and gender differences in the participants’ experiences were observed. Our findings indicate that the social distancing measures had several consequences for the mental and physical health and wellbeing of NH residents. In the theoretical framework described above, we outlined intended, unintended and unanticipated consequences as theoretical lenses and the foundation for our discussion and understanding of the consequences the social distancing measures had for NH residents during the first 9 months of the pandemic. This study contributes to the global literature of COVID-19 impacts on NH residents in particular in two ways. First, by providing new knowledge of such impacts from NHs in a universalistic welfare state context. Secondly, by interpreting the findings in light of the sociologist Robert Merton’s landmark work *“The unanticipated consequences of purposive action”* and De Zwart’s reinterpretation of Merton’s work. This theoretical background has helped us to differentiate 1) intended and anticipated consequences, 2) unintended, but not unanticipated consequences and 3) unintended and unanticipated, unforeseen and surprising consequences.

### Intended and Anticipated Consequences

In this study, all five NHs acted in accordance with the national policies and implemented stringent social distancing measures at the outbreak of the pandemic. The intended consequence of the measures was clearly to prevent COVID-19 being brought into the NHs and to hinder the spread between wards. The measures were effective in doing so, and three of the five NHs did not experience dissemination of infection or any infection at all during the first 9 months of the pandemic. In the same period, few NH residents became infected (3%) and died (1%) due to COVID-19 (Jacobsen et al., 2021), and this can be perceived as an intended and anticipated consequence of the social distancing measures as a national purposive measure. Despite Norway’s low infection rates, the share of COVID-19-related deaths in NHs, close to 50% during the first year of the pandemic (Jacobsen et al., 2021), is comparable to that of other countries. An international report from October 2020 showed that, of all COVID-19-related deaths in 20 countries, 46% were care home residents (Comas-Herrera et al., 2020). Further, the mortality rate in Norwegian NHs was high, and about one-third of the infected residents died (Jacobsen et al., 2021) indicating the frailty and vulnerability of residents (Thompson et al., 2020).

Altogether, 4 out of 10 COVID-19-related deaths in Norwegian NHs occurred in the 10 institutions with the most recorded deaths (Jacobsen et al., 2021). There are several possible explanations, and some NHs were particularly vulnerable, due to deficiencies in the buildings and challenges concerning the isolation of wards with infections from other wards and the establishment of cohorts of residents and staff. When a ward first contracted the infection, it was challenging to keep the infection down and prevent it from spreading between the residents and other wards. Part-time positions among staff and mobility between different workplaces might also explain why some NHs were particularly affected by high infection rates (Jacobsen et al., 2021). International research supports the assumption that facility-specific characteristics made some NHs more prone to the occurrence and spread of COVID-19 infections, for example, related to size and quality of buildings and to staffing (Ibrahim et al., 2021; Liu et al., 2020; Zimmerman et al., 2021).

We believe that the NHs in this study managed to prevent massive spread of COVID-19 in NHs and it reduced the number of deaths. Measures to curb the spread of the virus were of course intended, but it was an uncertain result in advance. Most NHs managed to keep their residents infection-free, while in those NHs who first contracted the infection were severely affected, due to the contagiousness of the disease and the residents’ susceptibility to this infection.

### Unintended, But Not Unanticipated Consequences

The study indicates that consequences of the social distancing measures led to social deprivation and loneliness among the NH residents. According to NH staff, many residents suffered from missing their families and social activities with other residents. This consequence can be perceived as unintended, but it is certainly not surprising or unanticipated. Several studies have investigated the links between social support, participation and activities with health outcomes for older adults. Similar to the results in our study, residents’ involvement in activities is found to foster social relationships, higher levels of connectedness and lower levels of loneliness (Drageset et al., 2011; Paque et al., 2018; Victor, 2012).

Our study has shown that visits from family members were of great importance to the health and wellbeing of residents. In a similar vein, international studies have documented that regular contact with family members helps residents to counteract decline in their health and level of functioning. Frequent visits also often support professional care with valuable and necessary information about the residents (Fry et al., 2015). In the aftermath of the pandemic, other studies have also documented that lack of social participation is associated with symptoms of depression (Egeljić-Mihailović et al., 2022), and increased anxiety and memory disorders were related to COVID-19 measures (Brooks et al., 2020; Paananen et al., 2021). While our data indicates some variation in how badly different groups of residents were affected, we argue that well-intended social distancing measures came at a high price. For example, NH staff described how residents with cognitive impairment struggled with confusion and how they failed to comply to isolation regimes. Moreover, they reported that social distancing measures also seemed to be very demanding for residents without cognitive impairment—and for those who enjoyed social activities and contact with family and friends prior to the pandemic.

Another unintended but probably not unanticipated consequence was the change in care provision. For example, the isolation routines resulted in limited time to carry out personal care tasks like showering and physical activities. Moreover, cases of less frequent medical attention from NH physicians were described. Nurses often replaced the physician in the medical visits and discussed observations and treatment interventions with the physicians by telephone. Additionally, several staff members and physicians reported a deterioration of the residents’ physical functioning, due to fewer physical activities and a temporary stop of physiotherapy services. This latter finding is consistent with previous research (Paananen et al., 2021). This study of the consequences of the social distancing measures has further shed light on the value and health benefit of movement and short daily walks for residents in NHs, and that the loss of daily physical activities negatively affected their health. Using Merton’s (2016) theoretical perspective as a lens, the consequences of reduced healthcare services and deteriorating physical health condition might be seen as something latent in relation to actions that were neither recognized nor intended.

Importantly, the social distancing measures issued by national authorities were followed systematically in all Norwegian NHs, independently of whether or not they had infected residents and independently of the infection rates in the municipalities. In hindsight, this universal approach seems to have been too bold and strict, not paying sufficient attention to the harmful consequences for the residents’ physical health and the suffering that social isolation caused. The visiting ban limiting access for relatives to visit their family members appears to have been very costly, especially for terminal residents who were restricted in having family members present as much as they wished for in the last days of their life.

### Unintended and Unanticipated, Unforeseen and Surprising Consequences

An unforeseen and surprising consequence of the restrictions was staff reporting some residents to be calmer and more relaxed when the wards closed. We interpret this to be positively related to more contact and interaction between residents and the ward staff in wards that initiated ward-based activities, and having the same staff present for longer periods of time. Previous studies have reported that the high turnover of staff and substitutes is stressful to residents (Bostick et al., 2006). The ward-based activities during this period of social distancing measures replaced activities open to all residents at the NH and seem to have facilitated better contact and communication between staff and residents. In a Canadian study, staff members felt that their relationship with the residents grew stronger during the pandemic. Augmented staff levels and staff carrying out activities previously performed by family caregivers provide opportunities for more contact and to get to know the residents in new ways (Hung et al., 2022).

Another explanation for residents becoming calmer could be that some residents might benefit from less environmental stimuli. In a recent study from the Netherlands, staff in NHs reported that residents with advanced dementia and persons with agitated or psychotic behavior benefited from the reduction in untargeted stimuli (e.g., noise) due to the COVID-19 restrictions. However, residents without dementia and people with depressive and apathetic behavior have been found to be more negatively affected by the reduction in this kind of stimulus (Knippenberg et al., 2022). These previous findings correspond to the results in our study.

Another surprising consequence of the social distancing measures is staff’s observations of changed visiting pattern in NHs. Staff have claimed that residents are getting fewer visits since the social distancing measures were ended. When the restrictions on visiting were lifted, families continued to stay away from the NHs or came for shorter visits than prior to the period with social distancing measures. The reasons for this are unclear, but it is likely that the way people think, feel and behave has been affected by these pandemic experiences, probably increasing their awareness of the spread of infections, making many more cautious concerning social actions that involve older vulnerable people. In a Norwegian study, approximately two out of five older persons 70 years or older reported their experience of social isolation. Half of them reported receiving fewer visits at home during the first year of the pandemic, particularly during the first and second infection waves. In addition, approximately half of the participants reported a fear of COVID-19 and were uncomfortable when they thought of the virus (Ibsen et al., 2022). It is thus reasonable to believe that some family members could be afraid of bringing infection into NHs or want to stay at home to protect themselves from being infected by others. Another study exploring the experiences of relatives of NH residents with COVID-19 found that family caregivers faced difficult dilemmas regarding visiting. Their risk of quarantine or of getting sick could have consequences for their ability to work, as well as for being together with other people important to them (Tretteteig et al., 2022).

By way of a preliminary conclusion, some of the consequences discussed are obvious and easily observable, like social distancing, decreasing level of physical and social activities, gradually declining mental health and level of physical functioning, and increased use of psychotropic drugs. Those consequences may be phrased as unintended but not unanticipated, while calmer and more relaxed residents, as described by the NH staff, may be seen as unintended and unanticipated. The interviews for this study revealed that initially latent consequences (reduced daily mobility for the residents), unnoticed by staff and family, increasingly became manifest as they became a conscious part of what they observed in the lives of residents.

The recent COVID-19 pandemic has highlighted the vulnerability of frail older adults residing in long-term care institutions internationally. The need for healthy aging in this population has been voiced by professionals and interest organizations alike, alluding to inadequate support systems during the pandemic, conditioned by both previous and newly emerging contextual factors. Supporting healthy aging in older adults in NHs and other residential care settings calls for a balance between disease control and securing meaningful social life. Nurses, in collaboration with other HCP, play a pivotal role in ensuring that NHs pay attention to both.

Knowledge of how social distancing measures during the COVID-19 pandemic also negatively impacted NH residents’ health and wellbeing should have implications for nursing and NH practices in later pandemics. It is important that managers of the municipal health and care services, NH managers, nursing home doctors and nurses carry out an evaluation of the social distancing measures and how these were handled. The aim should be to learn from how the measures were introduced and handled in NH during this pandemic. In this work, emphasis should be placed on evaluating measures that promoted the residents’ health and wellbeing as well as those that had unintended negative consequences for the residents’ health and wellbeing. National health and care authorities should have overall responsibility for the evaluation, and on this basis for developing a contingency plan for infection control in NHs that safeguard the health and wellbeing of residents in later pandemics.

### Methodological Considerations and Study Limitations

We found the qualitative exploratory design employing a case study approach (Yin, 2013), anchored in the epistemology of Gadamer’s philosophical hermeneutics (2004), to be an advantageous basis for this study as it gave us a sound foundation for investigating and understanding underlying processes of the distinct phenomenon we explored. Including a strategic selection of NHs helped us to obtain variation in the cases concerning and population size, infection pressure and incidence of COVID-19-related deaths. Investigating HCP’ perspectives made it possible to collect detailed descriptions of the phenomenon from a primary source of information close to the NH residents.

To enhance study trustworthiness, we have focused on strengthening research credibility, dependability, confirmability, transferability, and authenticity (Guba et al., 1994; Lincoln & Guba, 1985). As researchers we had no prior personal knowledge of the study participants. We have emphasized reflexive journaling during planning, data collection and data-analysis phases. As authors, we discussed in advance how the individual interviews and focus groups should be conducted. This helped us to develop a common understanding of how these should be carried out. The individual interviews were conducted through one-on-one conversations, where the researcher emphasized asking open-ended questions to the participant, as well as follow-up questions—which gave the researcher ample opportunity to go in depth on the topics explored. The focus group contributed to important data that gave broader understanding of the topics under investigation. We have had focused attention on careful documentation of study participants’ shared information, and on collecting sufficient descriptive data to ensure a thick description of participants’ observations and perspectives. We have highlighted to identify and portray observations and perspectives as expressed by the participants themselves. In all phases we have put emphasis on reflecting upon our own preunderstanding to prevent confirmation bias. Throughout the analysis we have sought after disconfirming evidence in the data before formulating our findings. In this process all findings have been considered as preliminary findings until we made our final conclusions of the study results. To propose a further understanding of underlying influences of the explored phenomenon we have used an appropriate and recognized theoretical framework to shed additional light on the empirical data. Finally, we have aimed to present a transparent documentation of our research process.

The study provides insight into HCP perspectives consequences of the social distancing measures for residents in five Norwegian NHs. However, several study limitations should be addressed. Within the limited timeframe allocated for participant recruitment, only five NHs were included. Moreover, only 26 staff, eight managers and five physicians were recruited. A larger and more divers sample of NH as well as HCP would most likely have added further depth to the data analysis. Restrictions in extending the recruitment period excluded this option. However, qualitative explorative studies can, even with small samples, generate new, in-depth understanding of phenomena that we currently have limited knowledge of (Brinkmann & Kvale, 2015). The finding reported from the study in this article does however only describe one perspective—the HCP perspective, and not the one of the NH residents themselves or their relatives. Future research should therefore explore and described also these latter first hands perspectives of those who actually experienced the social distancing measures and how it affected their health and wellbeing.

## Conclusion

Although social distancing measures were intended to protect residents from contracting COVID-19, our findings indicate that these measures came at a high cost for many NH residents. HCP observed that the measures also had a negative impact on the residents’ health and wellbeing. The pandemic led to new standards of care and measures that violated the standards not only of social care but also of holistic care. Several dilemmas related to the social distancing measures arose during this period. The main consequences for the residents found in this study were confusion and communication challenges, social deprivation and loneliness, and reduced healthcare services leading to deterioration of health condition.

Many of the consequences are not unanticipated—for example the cancellation of visits or activities resulted in loneliness and depression among residents. Interestingly, we also found unanticipated consequences in that some residents became calmer when the NHs locked down, pointing to the fact that the reduction of stimuli, and having the same staff present for longer shifts, positively impacted a segment of the residents. However, the elephant in the room is still how public health authorities implemented social distancing measures with few mitigation measures or the use of local discretion. For example, NHs with no infections were obliged to follow the same restrictions as NHs with much infection, and in many cases residents with short life expectancy were not allowed to have family members present as much as they would have liked in the last days of their lives.
